# A Survey of On-Call Ophthalmology Consultation Availability Across Emergency Rooms in Puerto Rico

**DOI:** 10.7759/cureus.90943

**Published:** 2025-08-25

**Authors:** Camille A Velez-Morell, Daniela V Martinez, Maria Diaz Rosario, Gabriel A Jimenez-Berrios, Lorena Montalvo

**Affiliations:** 1 Ophthalmology, University of Puerto Rico, San Juan, PRI; 2 School of Medicine, Universidad Central del Caribe, Bayamon, PRI; 3 Ophthalmology, University District Hospital, San Juan, PRI

**Keywords:** emergency ophthalmic care, emergency room, emergency room consultation, on-call ophthalmology, ophthalmology accessibility

## Abstract

Introduction

Ophthalmic emergencies pose a major challenge in emergency rooms (ERs), where timely access to specialized care is usually limited. However, most emergency departments (EDs) lack in-house ophthalmology services or the necessary diagnostic tools needed to manage acute eye conditions, which can potentially delay appropriate treatment. In Puerto Rico, limited access to ophthalmic care in some areas may lead patients to seek assistance in ERs for eye-related issues. This study aimed to evaluate the accessibility of ophthalmologic care in ERs across Puerto Rico by assessing the availability of on-call ophthalmology consultations during emergencies across hospitals on the island.

Methods

Based on a list of hospitals from the Puerto Rico Health Department website, a phone survey was conducted from September to October 2024, targeting EDs to determine the availability of on-call ophthalmology consults and protocols in place if such consults were unavailable. Hospitals specializing in specific services, those that declined participation, or those that were no longer in operation were excluded. Data were analyzed by island region and overall using descriptive and statistical analyses.

Results

Among the 43 EDs surveyed, 14 (32.6%) had an ophthalmologist on-call for emergencies, with six EDs (42.9%) offering 24/7 availability and eight (57.1%) part-time. Of these, six EDs (14.0%) reported that ophthalmologists saw emergency cases, while 37 EDs (86.0%) had on-shift doctors handle them. All EDs had emergency protocols, with 79.1% transferring acute cases. Additionally, 22 hospitals (51.2%) listed ophthalmology as an in-house service. Regional analysis showed significant variability in ophthalmology consult availability (p<0.0001), while no significant differences were observed between hospitals in terms of ophthalmologist consultations, 24/7 on-call availability, or part-time on-call availability (p>0.05).

Conclusions

Ophthalmology on-call consultation availability in EDs across Puerto Rican hospitals is limited, with availability being higher in the metro area. However, all hospitals included in this study have established protocols to manage ophthalmic emergencies. Our findings highlight the limited access to ophthalmologic care in Puerto Rican EDs, underscoring the need for improved protocols and resources.

## Introduction

In the United States, more than two million people visit the emergency room (ER) each year for eye-related complaints; however, it is still uncommon for emergency departments (EDs) to offer ophthalmology services [1]. Additionally, some EDs lack the equipment to make correct diagnoses [2], which could lead to a delay in appropriate treatment, leading to progression of disease and a state of irreversibility. While most visits can be attributed to non-emergent issues [3], ophthalmic emergencies like acute angle closure glaucoma, retinal detachments, lacerations, and compartment syndrome could lead to blindness if not treated promptly. Additionally, increasing wait times for outpatient ophthalmology clinic appointments could be driving a higher number of patients to seek immediate care in the ER [4].

Puerto Rico has a population of over three million people, and approximately only 165 ophthalmologists are available to serve this population [5,6]. As a result, patients with acute ophthalmic emergencies might opt for visiting the ER due to the lack of immediate availability of private practice or clinical appointments. Although it is rare for EDs to have an ophthalmologist [1] on-call, this study preliminarily aims to evaluate the accessibility of ophthalmologic care in Puerto Rican hospitals by surveying the availability of ophthalmology on-call consults for emergency room cases. The secondary aim is to explore whether there is a protocol in place for treating ophthalmic emergency cases when no ophthalmology consult is available, to ensure timely and appropriate treatment.

## Materials and methods

To assess the availability of ophthalmologic consults across Puerto Rican hospitals, a phone call survey was carried out from September to October 2024, targeting the EDs of hospitals listed on the official Puerto Rico Health Department (PRHD) website [7]. The publicly available phone numbers for the EDs were used, and informed consent was obtained upon contacting the appropriate party. The phone call survey consisted of six basic questions (Table 1) to quickly assess the availability of on-call ophthalmology consultation services in the EDs. Furthermore, EDs without on-call ophthalmology consults were asked if they had an established protocol in place for managing ophthalmic emergencies and who was responsible for handling these emergency cases.

**Table 1:** Phone survey questions

Survey
Is there an ophthalmologist on-call to consult during ophthalmic emergencies presenting to the emergency room?
If there is an ophthalmologist available, is the ophthalmologist on-call part-time or 24/7?
If no ophthalmologist is on-call, is there a protocol in place to manage the ophthalmic emergencies?
If no ophthalmologist is on-call, what specialist attends these cases in the emergency room?
If no ophthalmologist is on-call, is the patient transferred to another hospital?
If the patient is transferred, where are they transferred to?

In addition to the phone call survey, the publicly available website of each hospital was accessed to note if ophthalmology was listed as one of their in-hospital provided services. Hospitals were excluded if they specialized in specific services, such as cardiovascular centers or psychiatric facilities, declined to participate after providing informed consent, or were no longer in operation when called. As a result, 43 out of 66 EDs were included in this study. The data collected were analyzed according to island region, as per the PRHD website [7], and overall using descriptive analysis. To determine whether there were statistical differences in the availability of ophthalmology consults regionally or between hospitals, chi-squared analyses were performed. Additionally, a Fisher’s exact test was used to compare Metro areas with non-Metro areas. This study was performed after obtaining the IRB approval (protocol number: 2024-36).

## Results

**Figure 1:**
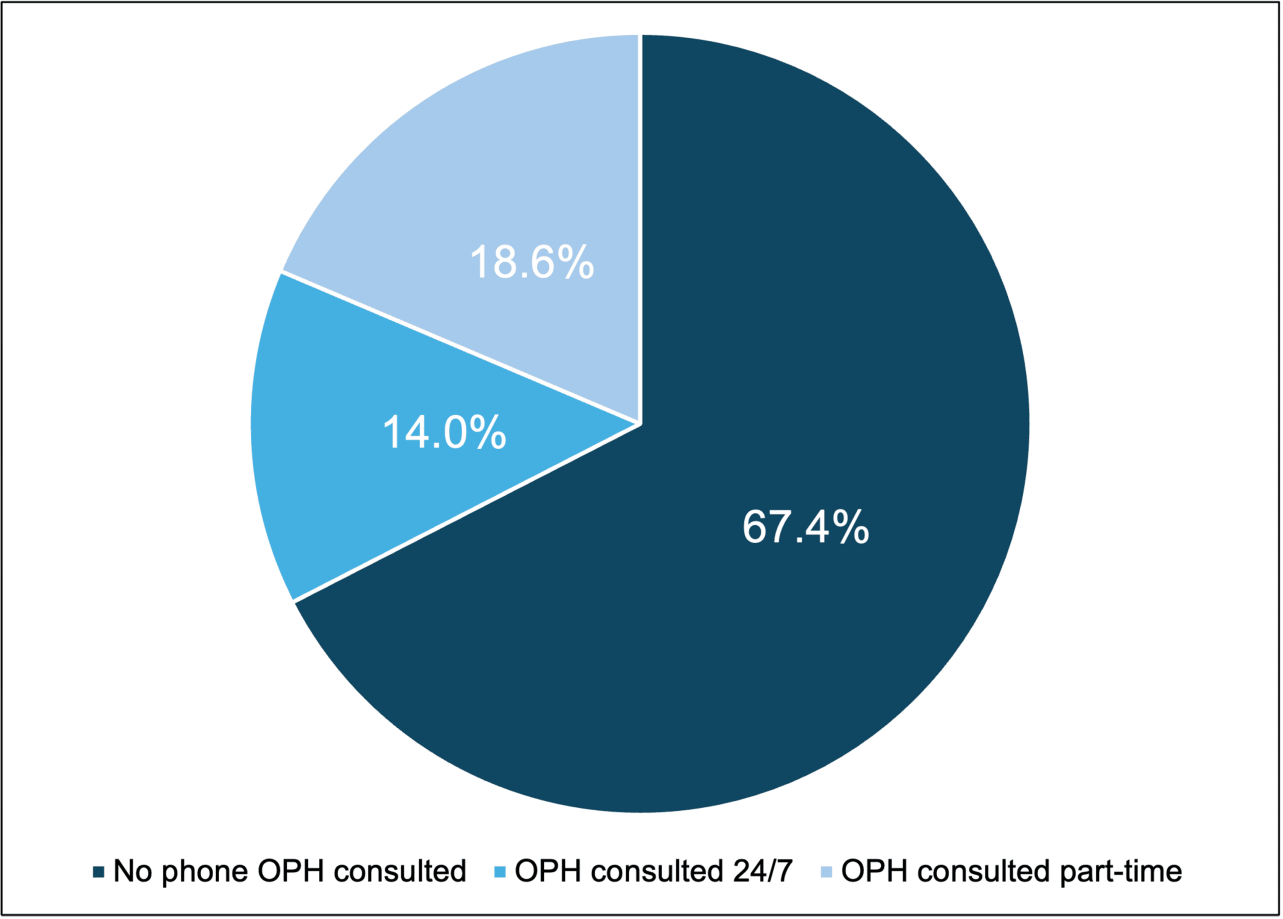
Ophthalmology on-call consult availability in emergency rooms across Puerto Rico OPH: ophthalmology

A total of 43 out of 66 EDs were included in this study (response rate: 65%). Of these 43 EDs, 14 (32.6%) reported consulting an ophthalmologist for ophthalmic emergencies compared to 29 (67.4%) that did not. Of the 14 EDs that consulted an ophthalmologist, six (42.9%) had the ophthalmologist on-call 24/7, and the remaining eight EDs (57.1%) had the ophthalmologist on-call part-time. Overall findings for on-call ophthalmologist consultation availability are summarized in Figure 1.

**Figure 2:**
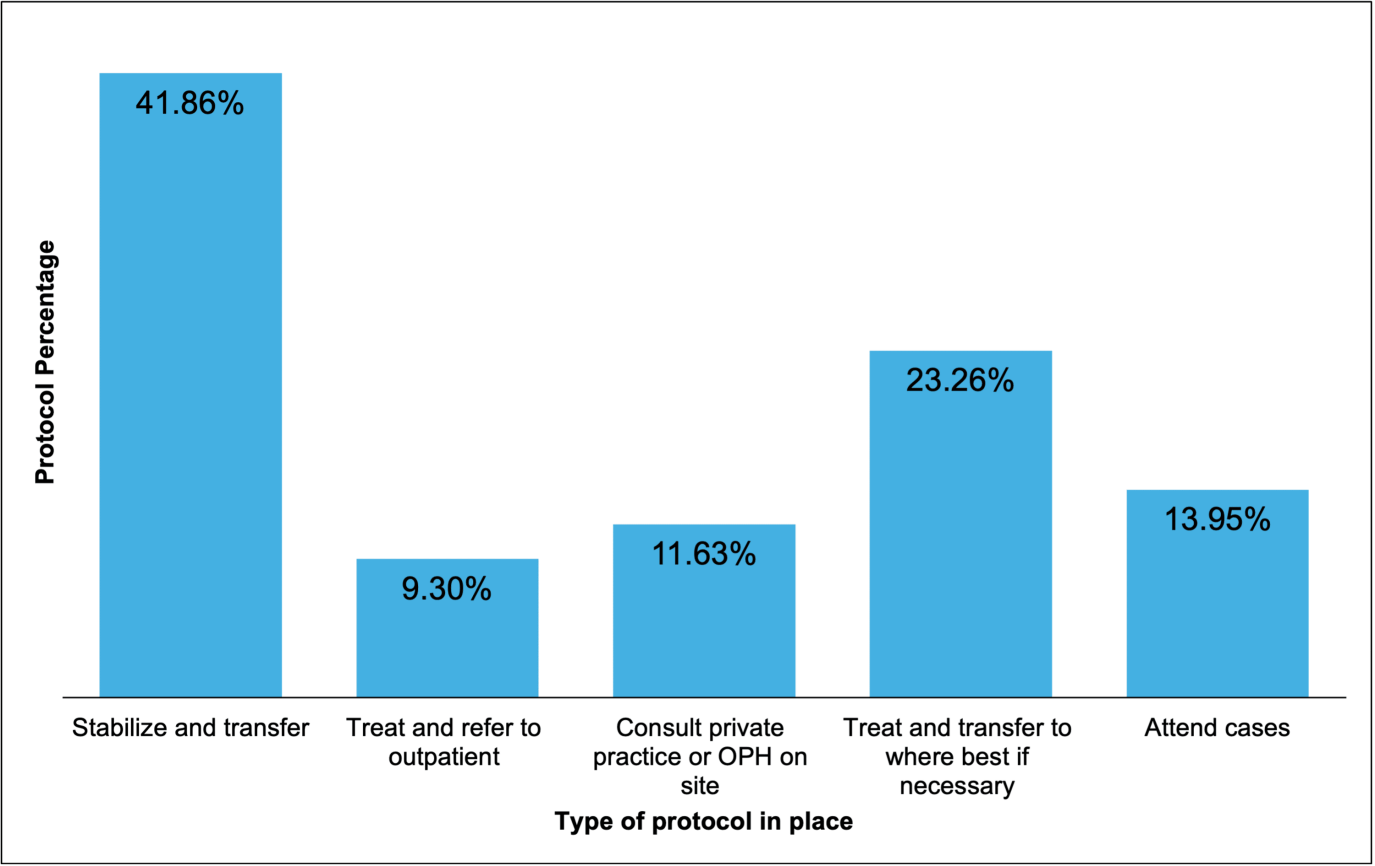
Types of protocols in place for ophthalmic emergencies Type of protocol established by emergency rooms across Puerto Rico to manage ophthalmic emergencies presenting to the emergency room

Even though 14 EDs consulted an ophthalmologist, only six EDs (14.0%) reported that emergency cases were eventually seen by an ophthalmologist on site, while the remaining 37 EDs (86.0%) reported that cases were addressed by the doctor on shift. All the EDs reported having an established protocol for managing ophthalmic emergencies; the specific protocol findings are summarized in Figure 2. When asked if patients were transferred to another facility, 34 EDs (79.1%) responded positively, with the majority transferring to the University Hospital. When investigating the hospital websites, 22 (51.2%) out of 43 hospitals listed ophthalmology as one of their available in-house services.

**Figure 3:**
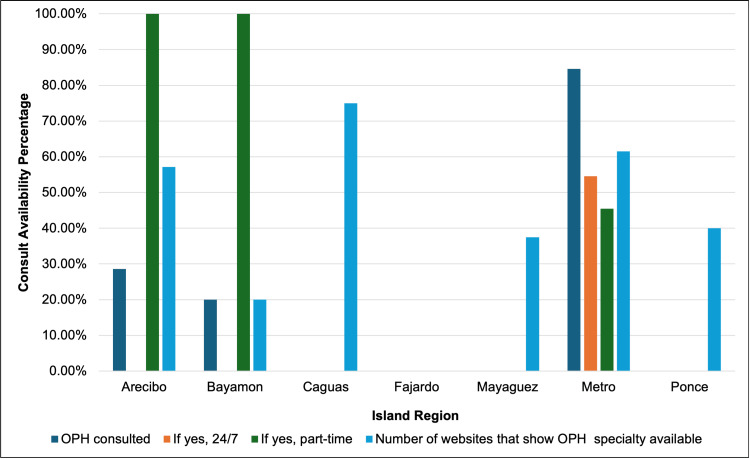
Ophthalmology on-call consult availability across emergency rooms in Puerto Rico by region The figure depicts ophthalmology on-call consult availability in emergency rooms across Puerto Rico according to regions as established by the official Puerto Rico Health Department (PRHD) website OPH: ophthalmology

To further understand how ophthalmology consults in EDs varied by regions, the data were analyzed according to the regions designated by the PRHD: Arecibo, Bayamon, Caguas, Fajardo, Mayaguez, Metro, and Ponce. Of note, 84.6% of Metro EDs consulted an ophthalmologist for ophthalmic emergencies compared to 28.6% of EDs in Arecibo and 20.0% of EDs in Bayamon. The findings according to regions are illustrated in Figure 3. Furthermore, statistical analysis using chi-squared tests (Table 2) revealed significant regional differences in the availability of ophthalmology consults (p<0.0001). Statistical differences were also found using Fisher's exact test when comparing the availability of ophthalmology consults in Metro vs. non-Metro areas (p<0.0001), as seen in Table 3. However, no significant differences were observed between hospitals in terms of ophthalmologist consultations, 24/7 on-call availability, or part-time on-call availability (p>0.05).

**Table 2:** Statistical analysis comparing OPH availability across regions and hospitals P-values for survey questions calculated using the Chi-square test (χ²). For 2x2 comparisons with low cell counts, Fisher’s exact test was used to confirm significance. Significance was assessed at α=0.05 OPH: ophthalmology

Division	Survey question	Percentage (n/N)	Chi-square value (χ²)	P-value	Significance
By region	Yes, OPH consult available	32.6% (14/43)	19.72	<0.0001	Yes
	Yes, 24/7 on-call OPH available	42.9% (6/14)	2.86	0.23 (>0.05)	No
	Yes, part-time on-call OPH available	57.1% (8/14)	1.07	0.30 (>0.05)	No
By hospital	Yes, OPH consult available	32.6% (14/43)	9.52	0.73 (>0.05)	No
	Yes, 24/7 on-call OPH available	42.9% (6/14)	0	1.0 (>0.05)	No
	Yes, part-time on-call OPH available	57.1% (8/14)	0	1.0 (>0.05)	No

**Table 3:** Metro vs. non-Metro area OPH consult availability Analysis of Metro versus non-Metro (all other regions) ophthalmology (OPH) consult availability. Odds ratios and p-values were calculated using Fisher’s exact test due to the small sample sizes of the data. An odds ratio of ∞ (infinity) indicates a complete separation in the data, where one group had no events in a specific category. Significance was assessed using α=0.05 OPH: ophthalmology

Metro area vs. non-Metro area	Odds ratio	P-value	Significance
Metro vs. non-Metro: OPH consult availability	49.5	<0.00001	Yes
Metro vs. non-Metro: 24/7 vs. part-time OPH availability	∞	0.209 (>0.05)	No

## Discussion

In this study, 32.6% of the surveyed EDs in Puerto Rico had an on-call ophthalmologist available for ophthalmic emergency consultations, which echoes findings of studies from the United States. Similarly, the majority (84.6%) of ophthalmology on-call consultations occurred in urban or metro area hospitals, which arguably serve a higher number of the Puerto Rican population compared to rural ones. The limited availability of ophthalmology consults could be attributed to the fact that most ophthalmic emergencies seen in high-volume emergency rooms are classified as low acute diagnoses with no risk for imminent loss of eyesight [3,8]. While there are limited studies profiling emergency room visits in Puerto Rico, the nature of the majority of ophthalmic emergencies might be similar; however, there are still ophthalmic emergencies that pose a threat, and a limited number of ophthalmologists allow same-day appointments [4]. Additionally, these ophthalmologists do not have adequate capacity to add new patients to their roster, which contrasts with the increasing demand from patients who wish to be seen at their convenience [4]. Thus, a higher number of patients visiting emergency rooms for non-emergent ophthalmic cases can overwhelm the number of resources available for truly emergent ophthalmic emergencies that are time-sensitive [9].

The fact that most of the ophthalmology on-call consultations were concentrated in metro areas highlights a disparity in access to specialized care between urban and rural regions. Patients living in rural areas may face additional barriers such as transportation limitations, fewer healthcare providers, and economic challenges that prevent them from receiving timely ophthalmic care. In fact, a recently published study highlights a higher proportion of rural patients compared to the number of available ophthalmologists serving rural areas [10]. This gap in services may be further contributing to health inequities, particularly for vulnerable populations.

One potential reason for these regional differences could be the geographic distribution of ophthalmologists, who are more likely to practice in urban areas where there are higher patient volumes, more private practice opportunities, and greater access to hospital infrastructure [10]. Rural hospitals may struggle to recruit or retain ophthalmologists due to fewer financial incentives, professional isolation, and lower patient volumes [11]. Additionally, metro area hospitals may have larger budgets and more robust emergency services, making it easier to offer specialized on-call services compared to smaller, resource-limited rural hospitals. Sociodemographic factors may also play a role, as rural populations often face higher rates of underinsurance or lack of health coverage, which may disincentivize specialists from offering services in these areas. Furthermore, rural hospitals may prioritize other specialties perceived as more urgent or broadly needed, leaving gaps in subspecialty services such as ophthalmology [12].

The potential rise in patients visiting the emergency room, coupled with the lack of appropriate equipment to diagnose ophthalmic emergencies [2] and the lack of on-call ophthalmology consultations, could lead to patients being misdiagnosed. A 2021 study found that 40% of ophthalmic-related presentations were misdiagnosed in the ED [13]. Therefore, having ophthalmology on-call services could ensure that patients receive appropriate treatment or stabilization before being transferred to another institution. Follow-up transition care could be facilitated if the ophthalmologist on-call agrees to follow-up treatment in their clinic [13]. Furthermore, 51.2% of the hospitals included in this study list ophthalmology as an in-hospital service on their website. Given this, EDs might benefit from leveraging ophthalmologists already on-site, further streamlining patient care by allowing consultations to better manage ophthalmic emergencies.

Even though most EDs in this study did not have on-call ophthalmologist consultations available, all EDs had a protocol in place for treating ophthalmic emergencies, with the majority being treated by the doctor on shift. Most EDs (41.86%) reported that they stabilized and transferred patients if necessary, while only a few EDs (9.30%) reported that they treated the patient and referred them to an outpatient clinic. Thus, patients with acute cases might receive the necessary care but may then be lost to follow-up if they are not being scheduled for follow-up care [13]. EDs could collaborate with nearby local ophthalmologists to refer patients for appropriate follow-up care. Furthermore, the outcome of time-sensitive ophthalmic emergencies like acute angle closure glaucoma, intraocular hemorrhages, and trauma could be affected by increased transportation times between hospitals due to patient transfers.

Given these findings, there are several interventions that could improve access to ophthalmic emergency care in Puerto Rico. EDs in rural areas could explore tele-ophthalmology services to allow remote consultation with specialists and reduce diagnostic errors. In addition, healthcare policymakers may consider developing incentive programs for ophthalmologists to provide on-call coverage, such as financial compensation or liability protections. Hospitals could also establish structured referral systems to ensure that patients with urgent ophthalmic conditions receive timely follow-up care. These interventions could help bridge the gap in access to ophthalmology services and improve patient outcomes.

Study limitations

Our data collection depended on a phone survey and a limited set of questions. The data collection strongly relied on whoever provided the information, which could impart some bias, including self-reporting bias, where hospitals may be overstating readiness or protocols. Additionally, a 65% response rate was obtained, which could affect findings compared to an 80-90% response rate. Information regarding in-hospital services offered by the hospitals was obtained from their websites, which may not be updated regularly or reflect actual services. Additionally, the study did not assess clinical outcomes or follow-up data, which limits our ability to determine the long-term impact of the lack of ophthalmology on-call services on patient care. Future studies could focus on evaluating patient outcomes, misdiagnosis rates, and the effectiveness of referral protocols, as well as incorporating other health services around the island to widen the sourcing of the sample data.

## Conclusions

Ophthalmology on-call consultation availability in emergency rooms across Puerto Rico is limited, reflecting similar trends observed on the mainland. However, all hospitals that agreed to participate in this study have protocols in place to address ophthalmic emergencies, ensuring effective and timely treatment. Availability of on-call ophthalmology services is higher in the metro area, while some emergency rooms rely entirely on stabilizing and transferring patients to other hospitals for appropriate care. Our findings highlight a potential need for on-call ophthalmologists in emergency rooms across Puerto Rico to effectively manage acute ophthalmic emergencies and ensure proper diagnosis and follow-up care. Additionally, further research is needed to gain deeper insights into the number of ophthalmic emergencies that present to emergency rooms in Puerto Rico and the impact that on-call ophthalmology availability can have on these patients’ outcomes.
